# Tip-Over Stability Analysis of a Pelvic Support Walking Robot

**DOI:** 10.1155/2020/1506250

**Published:** 2020-02-06

**Authors:** Yawei Han, Shuai Guo, Leigang Zhang, Fengfeng (Jeff) Xi, Weiwei Lu

**Affiliations:** ^1^Department of Mechatronic Engineering and Automation, Shanghai University, Shanghai, China; ^2^Department of Aerospace Engineering, Ryerson University, Toronto, Canada; ^3^Shanghai Testing & Inspection Institute for Medical Devices, Shanghai, China

## Abstract

Discussed in this paper is the tip-over stability analysis of a pelvic support walking robot. To improve the activities of daily living (ADL) in hemiplegic patients, a pelvic support walking robot is proposed to help patients facilitating their rehabilitation. During the gait training with the robot, the abnormal man-machine interaction forces may lead to the tip-over of the robot, which is not beneficial to the rehabilitation process. A new method is proposed to predict the possibility of tipping over and evaluate the stability of the robot based on statics model, dynamics model, and zero-moment point (ZMP) theory. Through the interaction forces and moments analysis with static case, the safe point (ZMP) is studied, and the influence factors of force/moment are analyzed by dynamics case. An optimization algorithm based on the genetic algorithm (GA) is proposed to reduce the risk of tipping over. The simulation results show that the optimization algorithm can keep the robot from tipping over when the interaction forces exceed the safety threshold.

## 1. Introduction

As a result of the acceleration of population aging, the World Health Organization reported over 17 million confirmed cases of stroke in 2016 [1]. Most stroke patients suffer from lower-extremity motor dysfunction after surgery, which severely affects the ADL of the patient. However, ∼70–80% of patients can benefit from the timely and effective rehabilitation process and can restore the motor function and balance function. During the gait training, the fall prevention and safe training environment are important. It is necessary to analyze the safety of the robot by the tip-over stability analysis.

In recent years, many universities, research institutes, and hospitals have developed mechanical equipment that can help the patients with the gait training. The Lokomat [2] is a highly automated suspension lower-limb rehabilitation robot that connects the lower-extremity exoskeleton system to the suspension weight reduction device through a four-bar mechanism. The robot is stationary and has little possibility of tipping over. The Andago [3] assisted rehabilitation robot developed by HOCOMA in Switzerland reduces the load on the lower limbs during the gait training by suspending weight loss on the pelvis. This suspension weight loss has a great influence on the coordinated movement of the upper and lower limbs. The designers avoid the tip-over by adding counterweight to the moving platform. The KineAssist [4] robot uses a pelvic support device with a moving platform to realize the movement of the patient, but the rotation center of the chassis is not on the same vertical line with the rotation center of the human body, directly affecting the patient's steering comfort. Carleton University developed a mobile limb training robot called GaitEnable [5]. This robot is smaller and lighter than KineAssist. It is a combination of a mobile lower-limb training robot and a walking robot. GaitEnable step trainer is omnidirectional. The device controls the support polygon of the robot, the position of the pelvis, and the posture of the robot. The GaitEnable and KineAssist are equipped with universal wheels to maintain stability via the change of the center of mass.

During the gait training, the man-machine interaction forces may exceed the normal values. There is a risk that the robot will tip over because the forces acting on the robot are beyond the safety threshold, causing potential harm to the patients. Based on this concerning, the tip-over stability of the robot should be analyzed to study the threshold of the interaction forces and the effective ways to stabilize the robot.

**Figure 1:**
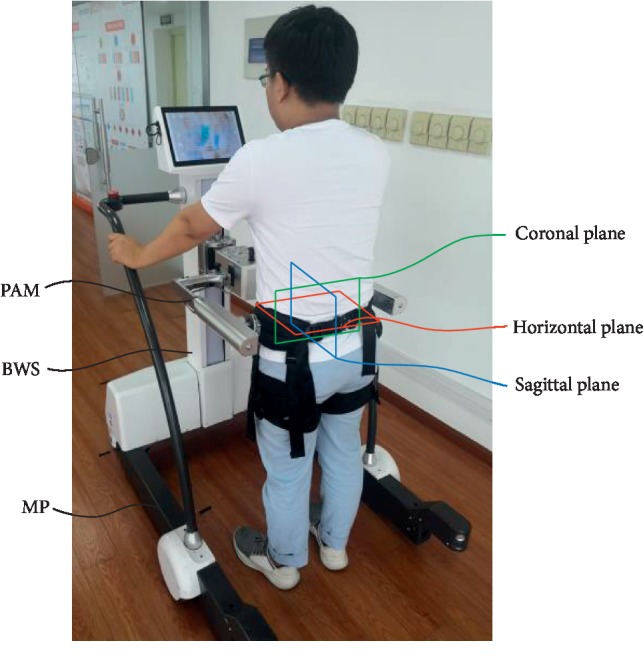
The components of the pelvic support walking robot. (a) The coordinate system. (b) The setting of the springs and sensors.

In this paper, the tip-over stability of a pelvic support walking robot, as shown in Figure 1, is analyzed: (1) the analysis of the safe range of interaction forces; (2) the analysis of the influence factors of tipping over; (3) the study of the optimization algorithm based on ZMP and GA.

## 2. System Description

**Figure 2:**
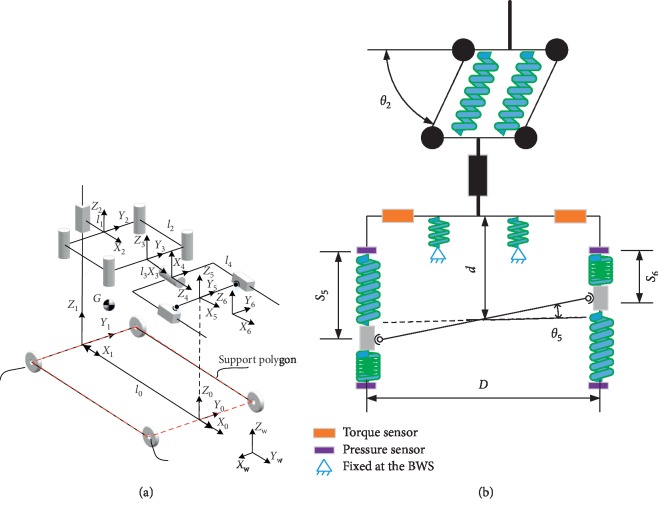
The schematic diagram of the mechanism.

The mechanical structure diagram of the robot is shown in Figure 2. Combining the revolute pair and the prismatic pair, the robot can realize the six degrees of freedom of motion in space: flexion and extension in the sagittal plane; adduction and abduction in the coronal plane; internal rotation and external rotation on the horizontal plane; and movement of the front, back, left, and right, and up and down. According the range of motion of healthy people [6, 7], the workspace of the robot is designed to be larger than the healthy people's need. Also, the rotation center of the robot should coincide with the people's rotation center to make the people more comfortable during the gait training.

The function of the robot is to provide the patients with pelvic support and help them during the gait training. This function is realized through the mobile platform (MP), the body weight support (BWS), and the pelvic-assisted mechanism (PAM). The PAM can support the patient and help them to realize the movement in cross section and the rotation around the sagittal axis, the vertical axis, and the coronal axis. The BWS can lift the pelvic support mechanism and patient to realize the movement in the vertical direction: the pelvis moves periodically along the vertical axis. With the help of the MP, the motion range of the patient can be extended throughout the environment.

As shown in Figure 2, the MP has two driving wheels installed on the rear to drive the mobile platform, and two universal wheels are installed at the front. With the differential motion of the two driving wheels, the robot can realize turning motion on the ground. And the robot can rotate around the center of two drive wheels' connection (around the *Z*-axis).

The BWS consists of a ball screw and a slider. The PAM is fixed at the slider, and the slider can move along the ball screw with low friction. The PAM can move along the *Z*-axis to adapt the motion of the pelvis.

The PAM provides support to the pelvis and can realize the motion of the pelvis. And the signals that needed to control the robot or evaluate the patient's movement are detected in this part.

The movement along the *X*-axis and *Y*-axis on the horizontal plane is realized by a four-bar mechanism as shown in Figure 2(b). The four-bar mechanism consists of four links. The rotation of *J*_2_ will produce the movement of *J*_3_ on the horizontal plane. A potentiometer is installed on *J*_2_ to detect *θ*_2_. The movement of *J*_3_ can be calculated: *X*=*l*_2_cos *θ*_2_, *Y*=*l*_2_sin *θ*_2_. In order to provide elasticity in the direction of *X*-axis and *Y*-axis, a spring damping mechanism is installed between the four links. The forces will overcome the movement along the *X*-axis and *Y*-axis and make the patient know whether their pelvis center deviates from the normal position. The elasticity also can be adjusted according to different patients' needs. The rotation of *J*_4_ realizes the rotation around the *X*-axis (the sagittal axis (α)). Springs are placed between *J*_4_ and the fixed base of the pelvic support mechanism to provide elasticity around the *X*-axis. *θ*_4_ can be detected by the potentiometer installed on *J*_4_. *P*_5_ and *P*_6_ are prismatic pairs. When *P*_5_ moves forward and *P*_6_ moves backward, the patient will realize the rotation around the *Z*-axis (the vertical axis (γ)). The sliders of *P*_5_ and *P*_6_ are located between springs to provide elasticity along the *Z*-axis. The pressure sensors detect the force from patient's rotation acting on the spring. By analyzing the forces, the direction of the rotation along the *Z*-axis can be studied. Also, the movement of *P*_5_ and *P*_6_ can be calculated through the force *F* and spring stiffness *k*: *s*=*F*/*k*. And *θ*_5_ can be calculated as: *θ*_5_≈(*S*_5_ − *S*_6_)/*D* when *θ*_5_ is small enough. Two universal joints are fixed at *P*_5_ and *P*_6_ to realize the rotation around the *Y*-axis (coronal axis (*β*)).

## 3. System Modeling and ZMP Theory

### 3.1. Statics Modeling

As shown in Figure 2, the robot coordinate system (*O*_0_*X*_0_*Y*_0_*Z*_0_) is fixed at the center of the connection of two driving wheels. The end-effector of the robot is the pelvis of patient, the *O*_6_*X*_6_*Y*_6_*Z*_6_ is attached. *O*_*i*_*X*_*i*_*Y*_*i*_*Z*_*i*_ (*i* = 1, 2,…, 5) is the joint coordinate system. The rotation center of the pelvis has to be consistent with the rotation center of the mechanism: *l*_0_=*l*_1_+*l*_2_+*l*_3_+*l*_4_; During the gait training, the parameter *Z*_1_ will change accordingly to realize the displacement in the *Z*-axis direction. The parallel four-bar mechanism composed of joints *J*_2_ and *J*_3_ realizes the *X*-axis and the displacement in the *Y*-axis direction: *X*=*l*_2_cos *θ*_2_, *Y*=*l*_2_sin *θ*_2_ (relative to the coordinate system ∑^ ^2). The establishment of the positive solution matrix equation is based on the recursive formula proposed by Xi et al., which is as follows [8]:

(1) $$\begin{array}{c} p_{i}=p_{0}+\overset{n}{\underset{i=1}{\sum }}R_{i}p_{i}^{\prime },\\ R_{i}=\overset{i}{\underset{j=1}{\prod }}R_{sj}R_{mj}, \end{array}$$

where

- **p**_*i*_ is the position of each joint relative to the world coordinate system;
- **p**_*i*_′ is the position vector;
- **R**_*i*_ is the rotation transformation matrix;
- **R**_*sj*_ is the coordinate transformation of the adjacent coordinate system;
- **R**_*mj*_ is the rotation around the current coordinate system, according to the mechanism diagram:

(2) $$\begin{array}{c} \left[\begin{array}{cc} R & P\\ 0 & 1 \end{array}\right]=\left[\begin{array}{cccc} C_{5}C_{6} & -S_{5} & C_{5}S_{6} & T_{1}\\ T_{2} & C_{4}C_{5} & T_{3} & l_{2}S_{2}\\ T_{4} & C_{5}S_{4} & T_{5} & Z_{0}\\ 0 & 0 & 0 & 1 \end{array}\right], \end{array}$$

where

*C*_2_=cos *θ*_2_,  *S*_2_=sin *θ*_2_, and so on; *T*_1_=*l*_0_ − *l*_1_ − *l*_3_ − *l*_2_*C*_2_ − *l*_4_; *T*_2_=*S*_4_*S*_6_+*C*_4_*S*_5_*C*_6_; *T*_3_=*C*_4_*S*_5_*S*_6_ − *S*_4_*C*_6_; *T*_4_=*C*_6_*S*_4_*S*_5_+*C*_4_*S*_6_; *T*_5_=*C*_4_*C*_6_+*S*_4_*S*_5_*S*_6_.

(3) $$\begin{array}{c} \left[\begin{array}{c} v_{x}\\ v_{y}\\ v_{z}\\ \omega_{x}\\ \omega_{y}\\ \omega_{z} \end{array}\right]=\left[\begin{array}{cccccc} 0 & -l_{2}S_{2} & 0 & 0 & 0 & 0\\ 0 & l_{2}C_{2} & 0 & 0 & 0 & 0\\ 1 & 0 & 0 & 0 & 0 & 0\\ 0 & 0 & 1 & 0 & 0 & 0\\ 0 & 0 & 0 & 0 & 0 & 1\\ 0 & 0 & 0 & \frac{1}{D} & -\frac{1}{D} & 0 \end{array}\right]\left[\begin{array}{c} {\dot{Z}}_{1}\\ {\dot{\theta }}_{2}\\ {\dot{\theta }}_{4}\\ {\dot{S}}_{5}\\ {\dot{S}}_{6}\\ {\dot{\theta }}_{6} \end{array}\right], \end{array}$$

where *D*=0.32 m, as shown in Figure 2, is the width of the mechanism, which hugs the pelvis.

The center of mass of the link can be viewed as at the center of figure of the link. The vector of the *i*th link centroid position in the ∑^ ^0: 

(4) $$\begin{array}{c} p_{ci}=p_{i-1}+\frac{1}{2}p_{i}=R_{i}p_{ci}^{\prime }. \end{array}$$

The centroid velocity of the *i*th link ${\dot{p}}_{ci}$ can be got by derivation on both sides of the equation:

(5) $$\begin{array}{c} {\dot{p}}_{ci}={\dot{R}}_{i}p_{ci}^{\prime }+R_{i}{\dot{p}}_{ci}^{\prime }=\omega_{i}\times p_{ci}+R_{i}{\dot{p}}_{ci}^{\prime }. \end{array}$$

The centroid acceleration of the *i*th link ${\ddot{p}}_{ci}$ can be got by derivation on both sides of equation (5):

(6) $$\begin{array}{c} {\ddot{p}}_{ci}={\dot{\omega }}_{i}\times p_{ci}+\omega_{i}\times {\dot{p}}_{ci}+{\dot{R}}_{i}{\dot{p}}_{ci}^{\prime }+R_{i}{\ddot{p}}_{ci}^{\prime }={\dot{\omega }}_{i}\times p_{ci}+\omega_{i}\times \left(\omega_{i}\times p_{ci}+R_{i}p_{ci}^{\prime }\right)+{\dot{R}}_{i}R_{i}^{T}R_{i}{\dot{p}}_{ci}^{\prime }+R_{i}{\ddot{p}}_{ci}^{\prime }={\dot{\omega }}_{i}\times p_{ci}+\omega_{i}\times \left(\omega_{i}\times p_{ci}\right)+2\left(\omega_{i}\times R_{i}p_{ci}^{\prime }\right)+R_{i}{\ddot{p}}_{ci}^{\prime }, \end{array}$$

where

- **ω**_*i*_ is the joint angular velocity of *i*th link;
- **p**_*ci*_ is the vector of the *i*th link centroid position in the ∑^ ^0; −${\dot{p}}_{ci}^{\prime }$ is the velocity of *i*th link;
- R_*i*_ is the transformation from ∑^ ^0 to ∑^ ^*i*.

### 3.2. Dynamics Modeling

As the movement of the pelvis has great influence on the robot's tip-over stability, it is necessary to establish a dynamics model to research the motion parameters' effect on the robot's risk of tipping over.

According to Xi et al., the force and moment acting on the *i*th joint:

(7) $$\begin{array}{c} \begin{array}{c} w_{i}=M_{i}{\dot{t}}_{iJ}+B_{i}+H_{ii+1}w_{i+1} \end{array}, \end{array}$$

where

- $w_{i}=\left[\begin{array}{cc} f_{iJ} & m_{iJ} \end{array}\right]\;$ consists of the force and moment of the *i*th joint;
- $M_{i}=\left[\begin{array}{cc} m_{i}1 & m_{i}{\tilde{p}}_{ic}^{T}\\ m_{i}{\tilde{p}}_{ic} & I_{i} \end{array}\right]$ is the generalized mass matrix of the *i*th link;
- ${\tilde{p}}_{ic}$ is the skew symmetric matrix of centroid vector of the *i*th joint;
- **I**_*i*_ is the inertia tensor of each link;
- ${\dot{t}}_{iJ}=\left[\begin{array}{c} {\ddot{p}}_{ci}-g\\ \alpha_{i} \end{array}\right]$ is the acceleration of the *i*th link;
- $B_{i}=\left[\begin{array}{c} m_{i}\omega_{i}\times \left(\omega_{i}\times p_{ic}\right)\\ \omega_{i}\times \left(I_{i}\omega_{i}\right) \end{array}\right]\;$ consists of the centrifugal force and gyro moment;
- *ω*_*i*_ is the angular velocity of the *i*th joint;
- **p**_*ic*_ is the vector pointing from the center of the *i*th link to the *i*th joint;
- $H_{ii+1}=\left[\begin{array}{cc} 1 & 0\\ {\tilde{p}}_{i} & 1 \end{array}\right]$ is the transformation matrix from *i* + 1th joint to *i*th joint;
- **p**_*i*_ is the vector pointing from joint *i* to the tip;
- ${\tilde{p}}_{i}$ is the antisymmetric matrix of **p**_*i*_.

All the forces and moments acting on the machine converted to joint *i* can be written as:

(8) $$\begin{array}{c} w_{i}=M_{i}{\dot{t}}_{iJ}+\overset{5}{\underset{k=i+1}{\sum }}\left(\overset{k}{\underset{j=k+1}{\prod }}H_{j-1,j}\right)M_{k}{\dot{t}}_{kJ}+B_{i}+\overset{5}{\underset{k=i+1}{\sum }}\left(\overset{k}{\underset{j=k+1}{\prod }}H_{j-1,j}\right)B_{k}+\left(\overset{5}{\underset{j=i+1}{\prod }}H_{j-1,j}\right)w_{p}, \end{array}$$

where *w*_*p*_ is the forces and moments between patient and robot.

### 3.3. ZMP Theory

The zero-moment point theory [9] is used to evaluate the tip-over stability by comparing the relative positional relationship between the ZMP and the support polygon. The component of the man-machine interaction forces and moments in the horizontal plane needs to be balanced by the friction and the friction torque:

(9) $$\begin{array}{c} \begin{array}{c} {\left(\overset{⟶}{OP}\times \overset{⟶}{R}\right)}^{H}+\overset{⟶}{OG}\times m_{s}g+M_{A}{}^{H}+{\left(\overset{⟶}{OA}\times F_{A}\right)}^{H}=0 \end{array}, \end{array}$$

(10) $$\begin{array}{c} \begin{array}{c} R+F_{A}+m_{s}g=0 \end{array}, \end{array}$$

where

- $\overset{⟶}{OP}$ is the vector from the ∑^ ^0 to the acting point of the friction force;
- $\overset{⟶}{R}$ is the friction force from the ground and can be calculated from equation (9);
- *G* is the center of gravity of the robot.

The force **F**_*A*_ and moment **M**_*A*_ equals to the forces and moments above the ∑^ ^0.

(11) $$\begin{array}{c} \begin{array}{c} R_{x}=-\left(F_{Ax}+m_{s}g_{x}\right) \end{array},\\ \begin{array}{c} R_{y}=-\left(F_{Ay}+m_{s}g_{y}\right) \end{array},\\ \begin{array}{c} R_{z}=-\left(F_{Az}+m_{s}g_{z}\right) \end{array}. \end{array}$$

The position of the point *P* (*P*_*x*_, *P*_*y*_) (zero-moment point) can be calculated as:

(12) $$\begin{array}{c} P_{x}=\frac{{\left(\overset{⟶}{OG}\times m_{s}g+M_{A}^{H}+{\left(\overset{⟶}{OA}\times F_{A}\right)}^{H}\right)}_{y}}{R_{z}},\\ P_{y}=\frac{{\left(\overset{⟶}{OG}\times m_{s}g+M_{A}^{H}+{\left(\overset{⟶}{OA}\times F_{A}\right)}^{H}\right)}_{x}}{R_{z}},\\ P_{z}=0. \end{array}$$

The support polygon is made up of a rectangle. Once the *P* is out of the rectangle, the robot will lose dynamic equilibrium and tip over.

## 4. System Simulation and Optimization

The simulations are carried out to analyze the possibility of tipping over of the robot. The input is man-machine interaction forces. These forces can be measured and calculated:

**F**_*x*_ is the force generated by the movement of the patient along the *X*-axis. **F**_*x*_ can be measured by the pressure sensors as shown in Figure 2(b).

**F**_*y*_ is the force generated by the movement of the patient along the *Y*-axis. **F**_*y*_ can be calculated as follows:

(13) $$\begin{array}{c} F_{y}=\Delta yK_{23}, \end{array}$$

where Δ*y* is the movement along the *Y*-axis, *K*_23_ is the spring damping stiffness between joint2 and joint3. *K*_23_ can be calculated through experiment. In this robot, *K*_23_=3.9 N/mm.

**F**_*z*_ is the force generated by the movement of the patient along the *Z*-axis. **F**_*z*_ can be measured and calculated by the torque sensors as shown in Figure 2(b).

(14) $$\begin{array}{c} F_{z}=\frac{T}{d}, \end{array}$$

where *T* is the torque measured by the torque sensors. *d* is the distance between the torque sensors and the center of the pelvis.

The support polygon is a rectangle: *L∗W*(1.03*∗*0.7 m). The coinciding of the round reaction force acting point (*P*) with the center of the rectangle can be seen as the safest condition of the robot. Evaluation function *Q* can describe the relation between the two points. If *P* coincides with the center of the support polygon, the function *Q* is 1. When *Q* is closer to 1, the robot is safer and less likely to tip over. When *Q* is closer to 0, the robot is more likely to tip over. If *P* exceeds the support polygon boundary, the function *Q* will be negative.

(15) $$\begin{array}{c} Q=\left\{\begin{array}{cc} \frac{\min\left(\left|P_{x}\right|,\left|P_{x}+\left(L/2\right)\right|\right)}{L}+\frac{\min\left(\left|P_{y}-\left(W/2\right)\right|,\left|P_{y}+\left(W/2\right)\right|\right)}{W}, & \text{when the ZMP}\left(P_{x},P_{y}\right)\text{ is inside the support polygon,}\\ -1, & \text{when the ZMP}\left(P_{x},P_{y}\right)\text{ is outside the support polygon}. \end{array}\right. \end{array}$$

### 4.1. Static Simulation

**Figure 3:**
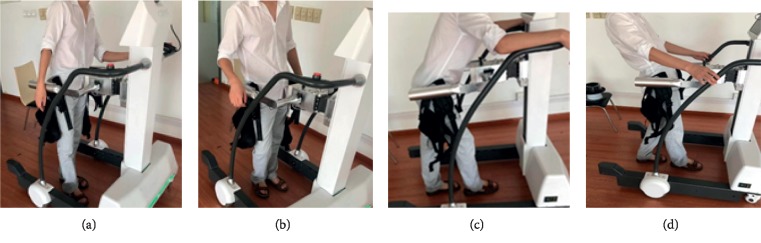
The four cases that the subject leans on the robot. (a) The subject leans on the machine to the left; (b) the subject leans on the machine to the right; (c) the subject leans on the machine to the forward; (d) the subject leans on the machine to the backward.

An experiment is designed to study the range of **F**_*x*_, **F**_*y*_, and **F**_*z*_, when the subject falls over and leans on the machine. As shown in Figure 3, there are four cases when the subject leans on the robot and the 8 subjects involved with different height and weight. The result is shown in Table 1. The static calculation model can be got by making $\omega_{i}=0,\;{\ddot{p}}_{ci}=0$, and **α**_*i*_=0 in the dynamic model. And so on, **B**_*i*_=0 and ${\dot{t}}_{iJ}=\left[\begin{array}{c} -g\\ 0 \end{array}\right]$. The force and moment acting on the ***i***th joint in the static model can be written as:

(16) $$\begin{array}{c} w_{i}=M_{i}{\dot{t}}_{iJ}+\overset{5}{\underset{k=i+1}{\sum }}\left(\overset{k}{\underset{j=k+1}{\prod }}H_{j-1,j}\right)M_{k}{\dot{t}}_{kJ}+\left(\overset{5}{\underset{j=i+1}{\prod }}H_{j-1,j}\right)w_{p}. \end{array}$$

**Figure 4:**
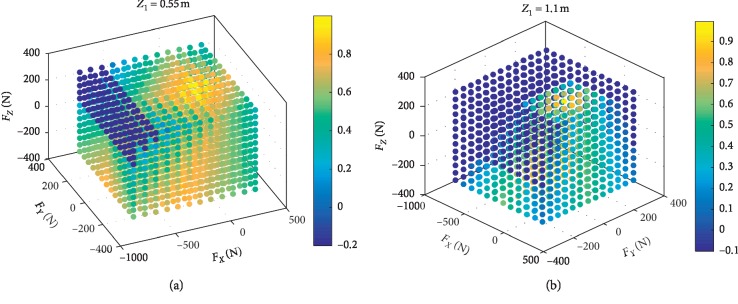
The relationship between the tip-over stability of the robot and the height of the pelvis: *Z*_1_. Each point represents an interaction force: **F**_xyz_. The points with light color mean the robot will not tip over, and the points with blue color mean the robot will tip over. (a) The number of the safe points is 1898 with the *Z*_1_ = 0.55 m. (b) The number of the safe points is 1225 with the *Z*_1_ = 1.1 m.

In the statics model, the parameter *Z*_0_ is the height of the pelvis, and the range of *Z*_1_ is from 0.55 m to 1.1 m. The simulations with different *Z*_1_ are carried out. The results in Figure 4 show that the higher the *Z*_1_ is, the more risk the robot with the same interaction force.

**Figure 5:**
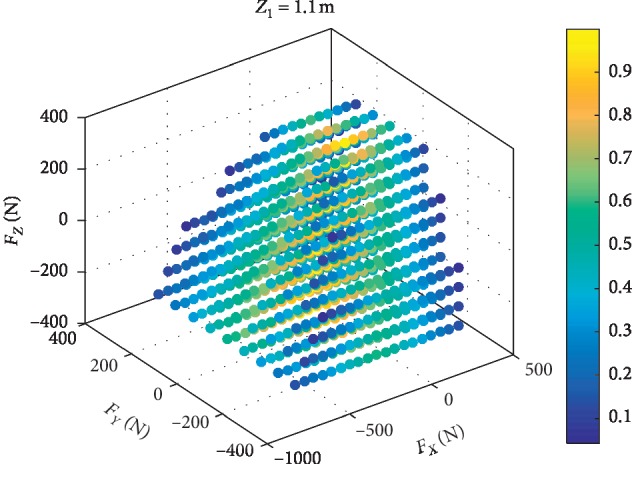
The safe range of *F*_*XYZ*_ with *Z*_1_=1.1 m.

In order to analyze the safe range of the input forces, make **F**_*x*_: −450 N∼300 N,  **F**_*y*_: −200 N∼300 N,  **F**_*z*_: −350 N∼350 N. Within the range of forces set, calculate *Q* with each force synthesized by **F**_*x*_, **F**_*y*_, and **F**_*z*_, and *Z*_1_=1.1 m. The calculation result is shown in Figure 5, and the force safety range: **F**_*XYZ*_=[**F**_*X*_, **F**_*Y*_, **F**_*Z*_]^*T*^  is as follows:

(17) $$\begin{array}{c} F_{XYZ}=\left\{\begin{array}{c} F_{Z}\leq 3.1F_{Y}+320,F_{Z}\leq 1.2F_{X}+180\\ \text{s.t. }0\leq F_{Y}\leq 200,0\leq F_{X}\leq 140,\\ F_{Z}\leq -3.1F_{Y}+320,F_{Z}\leq 1.2F_{X}+180\\ \text{s.t. }-200\leq F_{Y}<0,-400\leq F_{X}\leq 0. \end{array}\right. \end{array}$$

The points with light color means that with the effect of the forces the point represents, the ZMP: *P* is within the support polygon. If the color is blue, the robot will tip over and the patient is in danger.

The analysis of the tip-over stability in the static condition provides reference for the analysis with dynamics model. The impact factors such as **ω**_*i*_, **α**_*i*_, ${\ddot{p}}_{ci}$, and so on will be considered, and a control system based on genetic algorithm (GA) [10, 11] is used to optimize the tip-over stability of the robot.

### 4.2. Dynamic Simulation

The simulation with dynamic model considers the effect from the *ω*_*i*_(joint angular velocity) on the analysis of robot. The consideration of **ω**_*i*_ makes **I**_*i*_ (the inertia tensor of *i*th link) become an important influencing factor:

(18) $$\begin{array}{c} B_{i}=\left[\begin{array}{c} m_{i}\omega_{i}\times \left(\omega_{i}\times p_{ic}\right)\\ \omega_{i}\times \left(I_{i}\omega_{i}\right) \end{array}\right], \end{array}$$

(19) $$\begin{array}{c} M_{i}{\dot{t}}_{iJ}=\left[\begin{array}{cc} m_{i}1 & m_{i}{\tilde{p}}_{ic}^{T}\\ m_{i}{\tilde{p}}_{ic} & I_{i} \end{array}\right]\left[\begin{array}{c} {\ddot{p}}_{ci}-g\\ \alpha_{i} \end{array}\right]. \end{array}$$

**Figure 6:**
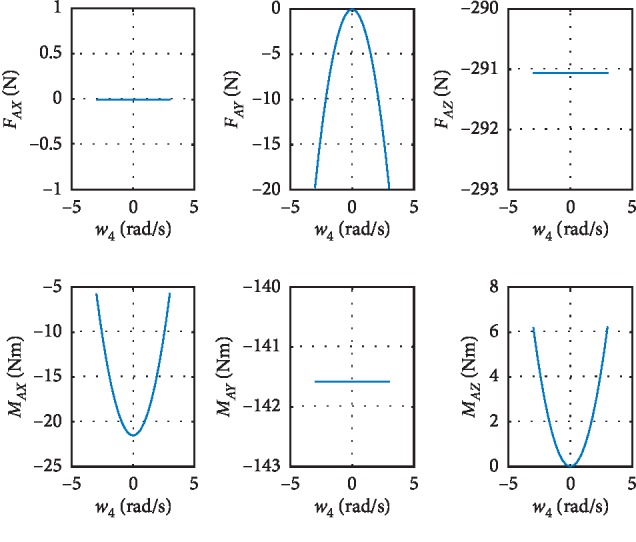
The influence of *ω*_2_ on the forces: *F*_*Ax*_,  *F*_*Ay*_ and *F*_*Az*_ and the moments: *M*_*Ax*_,  *M*_*Ay*_ and *M*_*Az*_.

**Figure 7:**
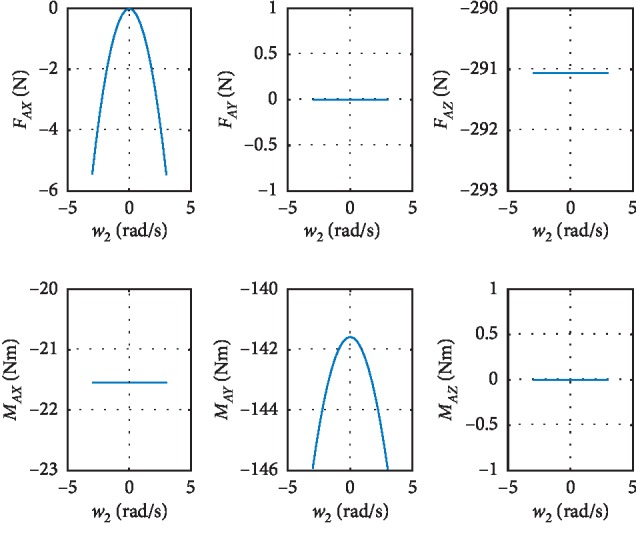
The influence of *ω*_4_ on the forces: *F*_*Ax*_,  *F*_*Ay*_ and *F*_*Az*_ and the moments: *M*_*Ax*_,  *M*_*Ay*_ and *M*_*Az*_.

As can be seen from equations (18) and (19), the larger the **ω**_*i*_ and **I**_*i*_ are, the larger the **ω**_*i*_ is. According to the quality of each link and the motion pattern of each joint, **ω**_2_ and **ω**_4_ may have great influence on the tip-over stability of the robot. The results of the simulation about **ω**_2_ and **ω**_4_ are shown in Figures 6 and 7. Although the variation range of *ω*_2_ and *ω*_4_ is four times than the normal value, the results show that *ω*_2_ and *ω*_4_ have little influence on the forces and moments of last joint.

### 4.3. Optimization

According to the analysis above, the height of the pelvis: *Z*_1_ has great influence on the tip-over stability of the robot. And other parameters such as *ω*_2_ and *ω*_4_ have little influence on the robot. Based on this, in the progress of the optimization, *Z*_1_ and the movement along *X*-axis: Δ*X* will be adjusted to look for the best combination of *Z*_1_ and Δ*X* to make *Q* nearest to 1.

The range of interaction force: **F**_*nxyz*_=[**F**_*nx*_, **F**_*ny*_, **F**_*nz*_] when healthy people walks with the robot normally can be obtained from the study by Ji et al. [12].

According to the analysis in the static simulation, the safe range of the interaction force: **F**_*X*_**F**_*Y*_ and **F**_*Z*_ is studied. Also, when the patient loses support and falls over, the interaction force: **F**_*xyz*_=[**F**_*x*_, **F**_*y*_, **F**_*z*_] is beyond the safe range. In order to make sure the robot is safer during using, safety factor *H* is proposed to calculate the safety threshold of the interaction force:

(20) $$\begin{array}{c} F_{XYZ}>F_{sxyz}=\frac{F_{XYZ}}{H}>F_{nxyz}. \end{array}$$

**Figure 8:**
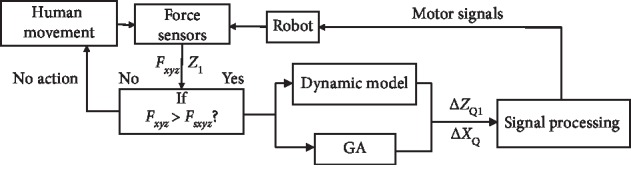
The control flow of the optimization algorithm.

An optimization algorithm based on genetic algorithm (GA) is proposed to prevent the robot from tipping over and make the evaluation function *Q* nearest to 1. The control flow chart is shown in Figure 8. When the interaction force is beyond **F**_*sxyz*_, GA will calculate the optimal Δ*Z*_1_ and Δ*X*. GA calculates the *Q* with different combinations of *Z*_1_ and Δ*X*, and chooses *Z*_*Q*1_ and Δ*X*_*Q*_ as the best combination when *Q* is the maximum. And the signals will be sent to the motors to adjust the height of the pelvic and the position of the support polygon.

(21) $$\begin{array}{c} \underset{Z_{Q1},\Delta X_{Q},Q}{\underset{︸}{\text{Maximize}}}\;\text{GA}\left(Z_{1},\Delta X,Q\right)\\ \text{s.t. }\;0.55\leq Z_{1}\leq 1.1,\\ -0.3\leq \Delta X\leq 0.3. \end{array}$$

### 4.4. Simulation

**Figure 9:**
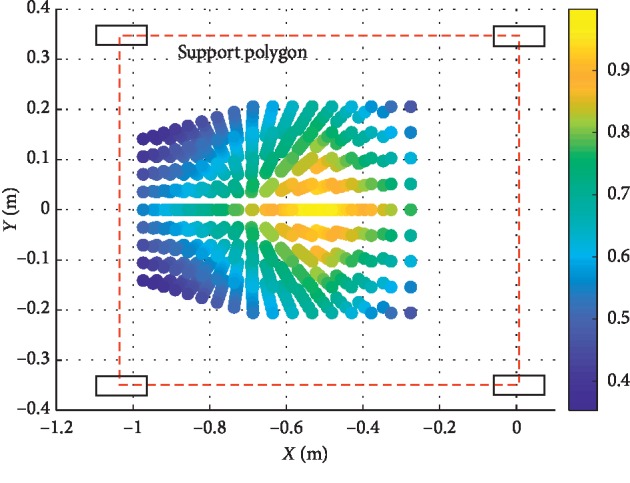
The distribution of *P*(ZMP) without the optimization algorithm.

**Figure 10:**
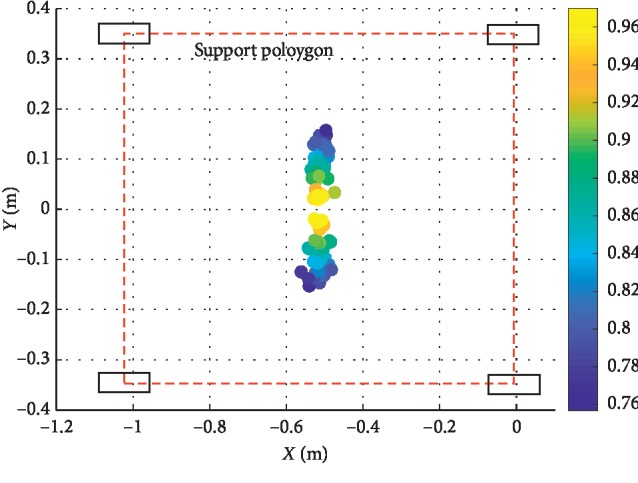
The distribution of *P*(ZMP) with the optimization algorithm.

In order to ensure the safety of the subject, a simulation is carried to instead of an experiment. The results are shown in Figures 9 and 10. In Figure 9, without the optimization algorithm, the distribution of the zero-moment points: *P* is throughout the support polygon. And in Figure 10, with the optimization, all the points are distributed around the center of the center of the support polygon.

### 4.5. Data Analysis and Discussion

The tip-over stability of the pelvic support walking robot is analyzed based on the statics model, the dynamics model, and the ZMP theory. Figure 3 shows the results of two simulations carried out with **F**_*x*_: −800 N∼300 N,  **F**_*y*_: −300 N∼300 N, **F**_*z*_: −350 N∼350 N. In Figure 4(a), with *Z*_1_=0.55 m, the most points' *Q* is positive with light color. The number of safe points is 1898. In Figure 4(b), with *Z*_1_=1.1 m, half of the points' *Q* is negative. The number of safe points is 1225. The results show that the height of the pelvis: *Z*_1_ has great influence on the tip-over stability of the robot. The safe range of the interaction forces is studied in Figure 5 with *Z*_1_=1.1 m. The safe range of **F**_*y*_ is symmetric around the *Y*-axis from −200 N to 200 N for that the robot is symmetry about the *xoz* plane. The safe range of **F**_*x*_ is from −400 N∼140 N for that the center of mass position is at the front of the robot. The safe range of **F**_*z*_ is a function of **F**_*x*_ and **F**_*y*_ as shown in (17). The influence of **ω**_2_ and **ω**_4_ is analyzed, and the results are shown in Figures 6 and 7. It shows that the influence of *ω*_2_ and *ω*_4_ is not so obvious, for that the max variation of **F**_*A*_ and **M**_*A*_ is 15 N and 20 N·m, respectively. The optimization based on the GA and the influence factors: *Z*_1_ and Δ*X* is proven to be effective in Figures 9 and 10. In Figure 9, without the optimization algorithm, the *P*(ZMP) is distributed throughout the support polygon. Some points' *Q* is close to 0.3, and the position is close to the boundary of the support polygon. With the interaction forces, these dangerous points represent the robot is likely to tip over. In Figure 10, the *Q* of these points is from 0.76 to 1, and the distribution of these points is limited to a rectangle: *X*: −0.58∼−0.48; *Y*: −0.16∼0.16. It means that with the optimization algorithm, the points whose interaction forces are beyond the safe range: **F**_*sxyz*_ are focused around the center of the support polygon with the adjustments of *Z*_1_ and Δ*X*.

## 5. Conclusions

The tip-over stability analysis of a pelvic support walking robot was introduced in this paper, and an optimization algorithm was proposed to optimize the tip-over stability of the robot. The influence of the height of the pelvis and the safe range of the interaction forces were studied through the simulation with statics model. With the dynamics model, it was proven that the joint angular velocity such as *ω*_2_ and *ω*_4_ have little influence on the forces and moments of the last joint. An optimization algorithm based on the dynamics model and the GA was proposed to optimize the tip-over stability when the interaction forces are beyond the safe range. Simulations were carried to verify the effectiveness of the optimization system. The follow-up work will be concentrated on the improvement of the optimization algorithm to reduce the running time of the algorithm. Also, the counterweight of the robot can be optimized to improve the tip-over stability.

## Figures and Tables

**Table 1:** The force along the *X*-axis and *Y*-axis when the patient falls over.

Subject	Height (cm)	Weight (kg)	+*F*_*x*_ (N)	−*F*_*x*_ (N)	+*F*_y_ (N)	−*F*_*y*_ (N)
S1	178	73	130	140	130	130
S2	170	78	160	165	155	130
S3	178	65	130	130	120	125
S4	173	55	90	90	85	75
S5	173	60	140	130	110	90
S6	173	62	100	110	100	110
S7	173	68	120	120	120	120
S8	169	63	130	140	100	110
Average	173	66	125	128	115	111

## Data Availability

The data used to support the findings of this study are available from the corresponding author upon request.

## Appendix

### Rotation Matrix

The rotation matrix referred in 3.1 kinematics modeling is shown as:

(A.1) $$\begin{array}{c} R_{s0}=\left[\begin{array}{ccc} 1 & 0 & 0\\ 0 & 1 & 0\\ 0 & 0 & 1 \end{array}\right];\;P_{0}^{\prime }=\left[\begin{array}{c} -l_{0}\\ 0\\ 0 \end{array}\right];\;R_{s1}=\left[\begin{array}{ccc} 1 & 0 & 0\\ 0 & 1 & 0\\ 0 & 0 & 1 \end{array}\right];\\ P_{1}^{\prime }=\left[\begin{array}{c} Z_{1}\\ 0\\ Z_{0} \end{array}\right];\;R_{s2}=\left[\begin{array}{ccc} 1 & 0 & 0\\ 0 & 1 & 0\\ 0 & 0 & 1 \end{array}\right];\;P_{2}^{\prime }=\left[\begin{array}{c} l_{2}C_{2}\\ l_{2}S_{2}\\ 0 \end{array}\right];\\ R_{s3}=\left[\begin{array}{ccc} 1 & 0 & 0\\ 0 & 1 & 0\\ 0 & 0 & 1 \end{array}\right];P_{3}^{\prime }=\left[\begin{array}{c} l_{3}\\ 0\\ 0 \end{array}\right];R_{s4}=\left[\begin{array}{ccc} 0 & 0 & 1\\ 1 & 0 & 0\\ 0 & 1 & 0 \end{array}\right];\;\\ R_{m4}=\left[\begin{array}{ccc} C4 & -S4 & 0\\ S4 & C4 & 0\\ 0 & 0 & 1 \end{array}\right];P_{4}^{\prime }=\left[\begin{array}{c} 0\\ 0\\ l_{4} \end{array}\right];R_{s5}=\left[\begin{array}{ccc} 1 & 0 & 0\\ 0 & 0 & 1\\ 0 & -1 & 0 \end{array}\right];\;\\ R_{m5}=\left[\begin{array}{ccc} C5 & -S5 & 0\\ S5 & C5 & 0\\ 0 & 0 & 1 \end{array}\right];P_{5}^{\prime }=\left[\begin{array}{c} 0\\ 0\\ 0 \end{array}\right];R_{s6}=\left[\begin{array}{ccc} 0 & 1 & 0\\ -1 & 0 & 0\\ 0 & 0 & 1 \end{array}\right]. \end{array}$$
